# Surface Toll-like receptor 3 expression in metastatic intestinal epithelial cells induces inflammatory cytokine production and promotes invasiveness

**DOI:** 10.1074/jbc.M117.784090

**Published:** 2017-07-17

**Authors:** Marit Bugge, Bjarte Bergstrom, Oda K. Eide, Helene Solli, Ingrid F. Kjønstad, Jørgen Stenvik, Terje Espevik, Nadra J. Nilsen

**Affiliations:** From the ‡ Centre of Molecular Inflammation Research, Department of Cancer Research and Molecular Medicine, Norwegian University of Science and Technology, 7489 Trondheim, Norway and; the § Clinic of Medicine, St. Olav's University Hospital, 7030 Trondheim, Norway

**Keywords:** cancer biology, cell surface, double-stranded RNA (dsRNA), innate immunity, interferon, intestinal epithelium, metastasis, Toll-like receptor (TLR), CXCL10/interferon γ-induced protein 10 (IP-10)

## Abstract

Toll-like receptors (TLRs) are innate immune receptors for sensing microbial molecules and damage-associated molecular patterns released from host cells. Double-stranded RNA and the synthetic analog polyinosinic:polycytidylic acid (poly(I:C)) bind and activate TLR3. This stimulation leads to recruitment of the adaptor molecule TRIF (Toll/IL-1 resistance (TIR) domain–containing adapter-inducing interferon β) and activation of the transcription factors nuclear factor κB (NF-κB) and interferon regulatory factor 3 (IRF-3), classically inducing IFNβ production. Here we report that, unlike non-metastatic intestinal epithelial cells (IECs), metastatic IECs express TLR3 and that TLR3 promotes invasiveness of these cells. In response to poly(I:C) addition, the metastatic IECs also induced the chemokine CXCL10 in a TLR3-, TRIF-, and IRF3-dependent manner but failed to produce IFNβ. This was in contrast to healthy and non-metastatic IECs, which did not respond to poly(I:C) stimulation. Endolysosomal acidification and the endosomal transporter protein UNC93B1 was required for poly(I:C)-induced CXCL10 production. However, TLR3-induced CXCL10 was triggered by immobilized poly(I:C), was only modestly affected by inhibition of endocytosis, and could be blocked with an anti-TLR3 antibody, indicating that TLR3 can still signal from the cell surface of these cells. Furthermore, plasma membrane fractions from metastatic IECs contained both full-length and cleaved TLR3, demonstrating surface expression of both forms of TLR3. Our results imply that metastatic IECs express surface TLR3, allowing it to sense extracellular stimuli that trigger chemokine responses and promote invasiveness in these cells. We conclude that altered TLR3 expression and localization may have implications for cancer progression.

## Introduction

Toll-like receptors (TLRs),^3^ Nod-like receptors (NLRs), and retinoic acid–inducible gene 1 (RIG-I)–like receptors (RLRs) are innate signaling pattern recognition receptors (PRRs) that are important for mounting an inflammatory response against invading microorganisms (1). PRRs recognize conserved microbe-associated molecular patterns expressed by microorganisms, but also sense damage-associated molecular patterns released from damaged host cells during injury (2). Activation of PRRs results in the production of inflammatory cytokines, type I IFNs, and initiation of innate and adaptive immune responses. Toll-like receptor 3 (TLR3) recognizes double-stranded RNA (dsRNA), a viral replication intermediate associated with virus infection. It also recognizes the synthetic dsRNA mimic polyinosinic:polycytidylic acid (poly(I:C)) and RNA released from necrotic cells as ligands (3, 4). Viral dsRNA and poly(I:C) are also recognized by TLR-independent pathways and trigger activation of the cytoplasmic RNA helicases RIG-I and Mda-5 in the cytosol (5–7).

PRR activation in cancer cells may contribute to the progression of cancer. Certain cancer cells up-regulate PRR expression and produce damage-associated molecular patterns that activate other cells in the tumor microenvironment, leading to further cytokine release and tumor-promoting inflammation (8). TLR3 activation induces inflammatory cytokines but also induces type I IFN production, which impairs proliferation and induces apoptosis in some cancer cells (9–11). IFNs additionally activate natural killer cells, enhance CD8+ T cell responses, and promote DC maturation (12). The ability to trigger type I IFNs has rendered TLR3 an attractive target in cancer therapy (12), but TLR3 has also been acknowledged as a mediator of pro-tumorigenic inflammation, which can drive cancer cell survival and proliferation (13). Thus, TLR3 signaling is multifaceted and may have opposing effects on cancer progression.

TLR3 is widely expressed in immune cells such as macrophages and myeloid dendritic cells (mDCs) but is also expressed in fibroblasts, neurons, and epithelial cells (14–17). The structure of TLR3 is similar to other TLR family members, consisting of a leucine-rich repeat domain, a transmembrane region, a linker region, and a Toll/IL-1 receptor (TIR) domain (18). The leucine-rich repeat domain mediates ligand binding, whereas the cytoplasmic TIR domain induces intracellular signaling. Binding of dsRNA induces TLR3 dimerization, recruitment of the adaptor molecule TIR domain–containing adapter-inducing interferon β (TRIF), and subsequent activation of the transcription factors nuclear factor κB (NF-κB) and interferon regulatory factor 3 (IRF3). This results in production of inflammatory cytokines and IFNβ (19). TLR3 is primarily expressed in the endoplasmic reticulum (ER) of resting mDCs but translocates to endosomes, upon stimulation, in a process dependent on the ER-resident transporter protein Unc-93 homolog B1 (UNC93B1) (20–23). TLR3 is further subject to proteolytic cleavage and posttranslational modifications in endolysosomal compartments (24–27). The binding of endocytosed dsRNA to TLR3 is influenced by endosomal acidity as well as dsRNA nucleotide length (28).

In this study we report that normal and cancerous intestinal epithelial cells (IECs) respond differently to TLR ligands. Cancerous IECs with metastatic potential expressed and up-regulated TLR3 and induced CXCL10 release in response to poly(I:C) addition in a TLR3-TRIF-IRF3–dependent manner. In contrast, healthy and non-metastatic IECs failed to respond to poly(I:C). Notably, metastatic IECs did not induce IFNβ in response to poly(I:C) addition and generally tolerated poly(I:C) exposure well. These cells expressed TLR3 in the plasma membrane, and CXCL10 release was observed in the absence of ligand internalization in response to low concentrations of poly(I:C), implying that TLR3 can signal from the cell surface of these cells. TLR3 activation also improved the invasive capacity of metastatic IECs. Our results imply that surface expression of TLR3 in metastatic IECs can promote the invasive capability of metastatic IECs and can induce chemokine responses to extracellular stimuli independent of ligand internalization.

## Results

### IEC lines display differences in CXCL8 and CXCL10 induction in response to TLR ligands

PRR stimulation may have different effects on the survival of tumor cells, depending on which PRRs they express and the signals they mediate. To investigate differences in TLR- and NLR-mediated signaling between cancerous and healthy IECs, we initially assayed TLR- and NLR-triggered cytokine responses in five generally available and well-characterized cancer IEC cell lines (HT29, HCT-116, SW480, SW620, and Caco-2) and the normal fetal IEC line FHC. We sought to compare TLR/NLR responses in cancer cell lines reported to display high metastatic ability *in vivo* (HT29, SW620, and HCT116 (29, 30)) with the poorly metastatic IECs (SW480 and Caco-2 (31, 32)) and healthy IECs (FHC). We were particularly interested in differences in TLR- and NLR-mediated responses in primary SW480 cells and their metastatic derivatives, SW620 cells (33, 34). The IECs were therefore assayed for a panel of cytokines (including TNF, IL-6, MIP-1β, MIP-1α, IL-1β, IL-12p70, CXCL8, CXCL10, and VEGF-A by ELISA) following challenge with the TLR2 ligands P_3_C and FSL-1, the TLR3 ligand poly(I:C), the TLR4 ligand LPS, and the NLR NOD2 ligand muramyl dipeptide (MDP) for 20 h.

**Figure 1.**
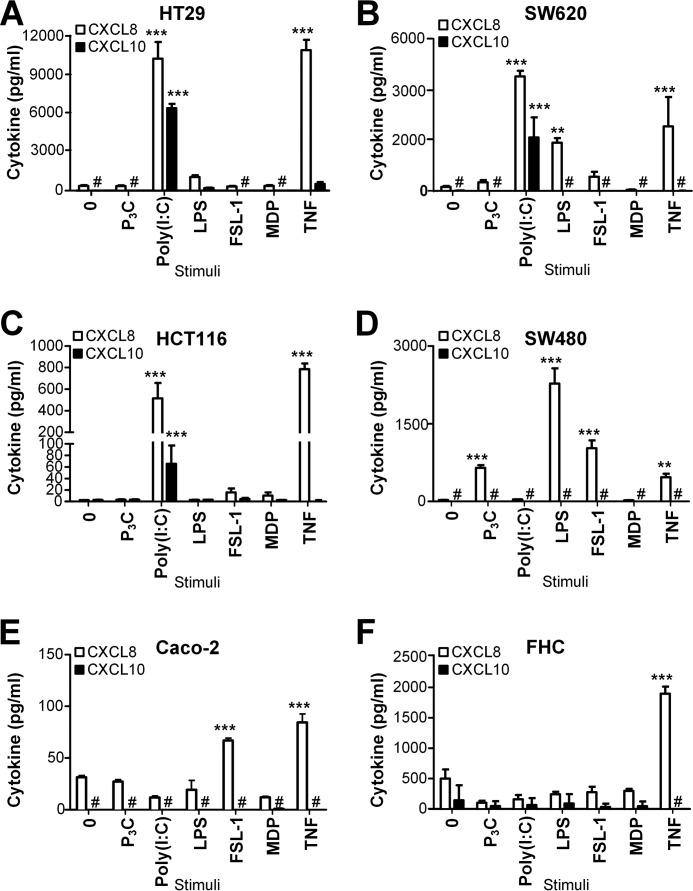
**IECs display differences in CXCL8 and CXCL10 release in response to TLR ligands.** *A–F*, the intestinal epithelial cell lines HT29 (*A*), SW620 (*B*), HCT116 (*C*), SW480 (*D*), Caco-2 (*E*), and FHC (*F*) were treated with medium (*0*), P_3_C (100 ng/ml), poly(I:C) (50 μg/ml), LPS (100 ng/ml), FSL-1 (100 ng/ml), MDP (1 μg/ml), and TNF (100 ng/ml) for 20 h before supernatants were harvested and assayed for CXCL8 and CXCL10 content by ELISA. The results are presented as mean ± S.D. of triplicates and are representative of a minimum of two experiments. ***, *p* < 0.001; **, *p* < 0.01 *versus* medium (one-way ANOVA, Bonferroni post-test).

We observed CXCL8 release in several of the cell lines in response to the TLR2 ligands P_3_C and FSL-1, the TLR3 ligand poly(I:C), and the TLR4 ligand LPS following 20 h of stimulation (Fig. 1). No CXCL8 induction was observed in any of these IECs in response to the TLR7/8 ligand R848, the TLR9 ligand CpG, or a NLR NOD1 ligand (iE-DAP dipeptide) (data not shown). Non-cancerous IECs (FHC) did not induce CXCL8 production in response to any of the TLR or NLR ligands tested (Fig. 1*F*). These cells still produced CXCL8 in response to TNF (Fig. 1*F*), demonstrating that these cells do induce CXCL8 production in response to inflammatory cytokines. Interestingly, only the highly metastatic cell lines SW620, HT29, and HCT116 induced CXCL8 in response to the TLR3 ligand poly(I:C) (Fig. 1, *A–C*), whereas the poorly metastatic SW480 and Caco-2 cells failed to do so (Fig. 1, *D* and *E*). In contrast to SW620 cells, which responded potently to poly(I:C) (Fig. 1*B*), SW480 cells failed to induce CXCL8 in response to poly(I:C), even at high concentrations (50 μg/ml) of ligand (Fig. 1*D*). We found this particularly interesting because the cell lines SW480 and SW620 were originally isolated from the same patient (33). The three metastatic IEC cell lines SW620, HCT116, and HT29 also induced potent CXCL10 production in response to poly(I:C) addition (Fig. 1, *A–C*), whereas SW480 and Caco-2 cells failed to release detectable levels of CXCL10 (Fig. 1, *D* and *E*). No detectable TNF, IL-6, MIP-1β, MIP-1α, IL-1β, IL-12p70, or VEGF-A was observed in supernatants from IECs stimulated with any of the tested TLR or NLR ligands (data not shown). Combined, these results indicate that healthy primary IECs respond poorly to TLR stimulation compared with cancerous IECs and that metastatic IECs are particularly responsive to poly(I:C) stimulation.

### Poly(I:C)-responsive IECs up-regulate TLR3 expression and induce CXCL10 in a TLR3- and TRIF-dependent manner

**Figure 2.**
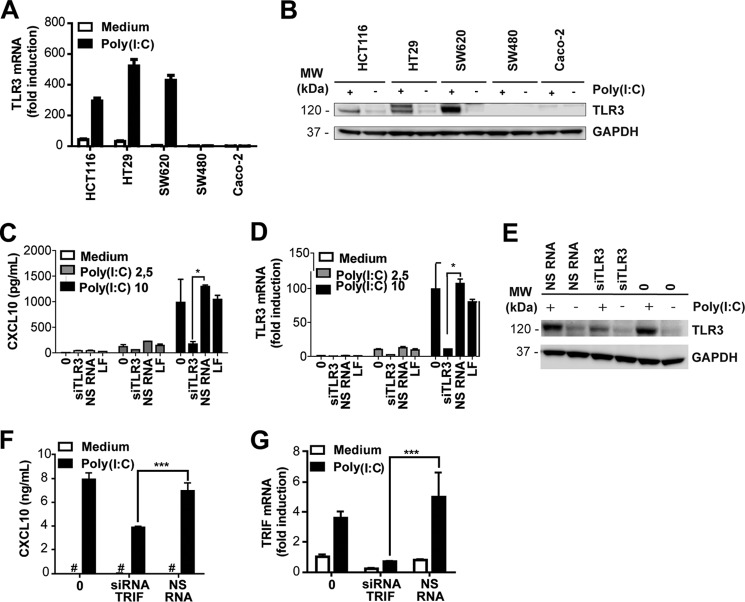
**Poly(I:C)-responsive IECs up-regulate TLR3 expression and induce CXCL10 in a TLR3- and TRIF-dependent manner.** *A*, TLR3 mRNA expression in SW620, SW480, HT29, HCT116, and Caco-2 cells treated with medium or poly(I:C) (5 μg/ml) for 24 h before TLR3 expression was determined by qPCR. GAPDH served as an internal control. The results show mean induction of triplicates ± S.D. relative to untreated Caco-2 cells and are representative of three independent experiments. *B*, TLR3 protein expression in SW620, SW480, HT29, HCT116, and Caco-2 cells left untreated (−) or treated with poly(I:C) (2.5 μg/ml) (+) for 24 h. Western blots were stained with anti-TLR3 (*top*) or GAPDH as a loading control (*bottom*). *MW*, molecular weight. *C* and *D*, CXCL10 release (*C*) and TLR3 mRNA expression (*D*) in SW620 cells left untreated (*0*), transfected with TLR3 siRNA (*siRNA TLR3.5*, 5 nm) or NS RNA (5 nm) or treated with the transfection reagent LF alone for 28 h prior to stimulation with poly(I:C) (2.5 or 10 μg/ml) for 20 h. CXCL10 release in the supernatant was assessed by ELISA. TLR3 mRNA expression in the lysates of the treated SW620 cells was determined by qPCR using GAPDH as an internal control. Results show mean ± S.D. of triplicates and are representative of three independent experiments. *E*, Western blots showing TLR3 (*top*) and GAPDH (*bottom*) expression in SW620 cells transfected with TLR3 siRNA (10 nm), NS RNA (10 nm), or transfection reagent (0) alone for 24 h prior to stimulation with poly(I:C) (2.5 μg/ml) for 24 h. *F* and *G*, CXCL10 production (*F*) and TRIF mRNA expression (*G*) in HT29 cells left untreated (*0*) or treated with siRNA against TRIF (20, 15, 10, or 5 nm) or with NS RNA (10 nm) for 24 h prior to stimulation with poly(I:C) (5 μg/ml) for 20 h. CXCL10 content in cell supernatant was assessed by ELISA, whereas silencing of TRIF was confirmed by assessing TRIF mRNA by qPCR using GAPDH as a reference control. The results show mean ± S.D. of triplicates and are representative of two independent experiments. ***, *p* < 0.001 *versus* NS RNA (one-way ANOVA, Holm-Sidak multiple comparisons).

Poly(I:C) is sensed by TLR3 as well as by the cytosolic RNA helicases RIG-I and Mda-5 when it is localized to the cytosol, *e.g.* by means of transfection. Because we observed that the IECs SW620, HCT116, and HT29 induced CXCL10 release upon addition of poly(I:C) in the absence of transfection reagent, we hypothesized that this response was mediated by TLR3. We initially quantified TLR3 mRNA in IECs in the absence and presence of poly(I:C) stimulation to determine whether TLR3 expression is regulated in response to stimuli in these cells. The metastatic IECs HCT116, HT29, and SW620 up-regulated TLR3 mRNA in response to poly(I:C) (Fig. 2*A*) in comparison with SW480 and Caco-2 cells (Fig. 2*A*). Notably, TLR3 expression was not up-regulated in SW480 cells in response to poly(I:C) stimulation but was strongly up-regulated in their metastatic derivatives, SW620 cells, following poly(I:C) treatment (Fig. 2*A*). We verified TLR3 expression in IECs at the protein level by staining Western blots of IEC lysates for TLR3. TLR3 protein expression was markedly up-regulated in SW620 cells in response to poly(I:C), whereas TLR3 expression was undetectable in SW480 or Caco-2 cells (Fig. 2*B*). HCT116 and HT29 cells expressed TLR3 protein and further up-regulated expression following poly(I:C) stimulation (Fig. 2*B*). These results were consistent with the results obtained at the mRNA level (Fig. 2*A*). TLR3 expression in the metastatic IECs SW620, HCT116, and HT29 parallels their ability to induce CXCL8 and CXCL10 in response to poly(I:C) addition, suggesting that this response may be mediated by TLR3.

We proceeded to confirm the role of TLR3 in mediating poly(I:C)-induced CXCL10 by silencing TLR3 with siRNA. We have shown previously that CXCL10 production is impaired in HT29 cells in response to poly(I:C) addition upon silencing of TLR3 with siRNA (35). To determine whether this is the case in SW620 cells as well, we treated these cells with siRNA against TLR3 (TLR3.5) or a non-silencing siRNA (NS RNA) prior to addition of poly(I:C) for 20 h. The supernatant was subsequently analyzed for CXCL10 content, whereas cell lysates were assayed for TLR3 expression by quantitative real-time PCR (qPCR). Cells treated with siRNA against TLR3 displayed impaired CXCL10 release in response to poly(I:C) (Fig. 2*C*) and expressed significantly less TLR3 mRNA (Fig. 2*D*) compared with cells treated with NS RNA (Fig. 2, *C* and *D*). This demonstrates that CXCL10 induction in response to poly(I:C) treatment is mediated by TLR3 in these cells. The efficiency of silencing TLR3 expression in SW620 cells with TLR3 siRNA was further verified at the protein level in poly(I:C)-stimulated SW620 cells (Fig. 2*E*). Cell viability was unaffected by siRNA treatment or poly(I:C) stimulation, and similar levels of silencing of TLR3 mRNA were observed with two siRNAs, TLR3.5 and TLR3.8 (data not shown). These results verify that SW620 cells express TLR3 and that CXCL10 production induced by poly(I:C) addition is TLR3-dependent in these cells.

TLR3 signaling requires the signaling adaptor protein TRIF/TICAM-1 (36). Because we observed that CXCL10 induction in metastatic IECs was mediated by TLR3 in response to poly(I:C) addition (Fig. 2*C* and Ref. 35), we proceeded to determine the role of TRIF in mediating this response. Poly(I:C)-responsive HT29 cells were left untreated or treated with siRNA against TRIF or non-silencing siRNA prior to stimulation with poly(I:C) (5 μg/ml) for 20 h. CXCL10 release in the cell supernatant was assayed by ELISA and was found to be significantly impaired in cells treated with siRNA against TRIF (Fig. 2*F*). Efficient silencing of TRIF was confirmed by assessing the mRNA expression of TRIF in samples by qPCR (Fig. 2*G*). Similar results were obtained in the poly(I:C)-responsive IEC cell line SW620 (data not shown). These results imply that TRIF is required for CXCL10 induction in response to poly(I:C) in metastatic IECs and support a role for TLR3 in mediating this response.

### IECs induce IFNβ in response to transfected poly(I:C) but not in response to addition of poly(I:C)

**Figure 3.**
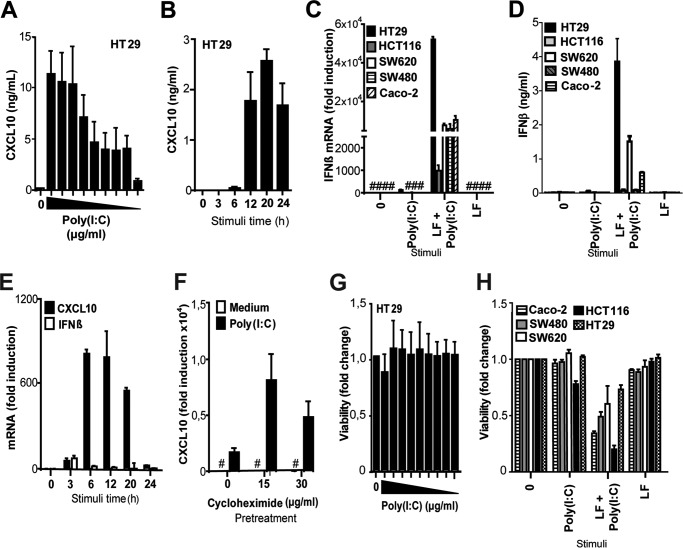
**IFNβ is induced in IECs in response to transfection with poly(I:C) but not in response to poly(I:C) addition.** *A*, HT29 *cells* were left untreated (*0*) or stimulated with poly(I:C) (50, 25, 10, 5, 2.5, 1.25, 0.63, 0.31, and 0.15 μg/ml) for 20 h before CXCL10 in the supernatant was assessed by ELISA. *B*, kinetics of CXCL10 release assessed by ELISA in supernatant from HT29 cells stimulated with poly(I:C) (2.5 μg/ml) for 0, 3, 5, 12, 20, and 24 h. The results are presented as mean ± S.D. of triplicates. *C*, IFNβ mRNA induction in HT29, HCT116, SW620, SW480, and Caco-2 cells treated with poly(I:C) (2 μg/ml) alone (*Poly(I:C)*), transfected with poly(I:C) complexed with Lipofectamine RNAimax (*LF* + *Poly(I:C)*, 2 μg/ml), or treated with only Lipofectamine RNAimax (*LF*) for 20 h. IFNβ mRNA induction was determined by qPCR. The results are presented as relative induction compared with medium-treated Caco-2 cells. GAPDH served as an internal control. Results show mean -fold induction ± S.D. of triplicates. *D*, IFNβ protein production in HT29, HCT116, SW620, SW480, and Caco-2 cells treated with poly(I:C) (2 μg/ml) alone, transfected with poly(I:C) complexed with Lipofectamine RNAimax (2 μg/ml), or treated with only Lipofectamine RNAimax for 20 h. IFNβ in the supernatant was assessed by ELISA, and the results show mean ± S.D. of three samples. *E*, HT29 cells were stimulated with poly(I:C) (2.5 μg/ml) for 0, 3, 6, 12, 20, or 24 h before CXCL10 and IFNβ mRNA induction was determined by qPCR. The results show relative induction with a non-treated sample as reference. GAPDH served as an internal control. The results show mean -fold induction ± S.D. of triplicates. *F*, CXCL10 mRNA induction in HT29 cells pretreated with cycloheximide (0, 15, or 30 μg/ml) for 30 min prior to stimulation with poly(I:C) (2.5 μg/ml) for 8 h. CXCL10 mRNA was determined by qPCR (normalized to medium control and the endogenous control TBP). *G*, viability in HT29 cells left untreated (*0*) or stimulated with poly(I:C) (50, 25, 10, 5, 2.5, 1.25, 0.63, 0.31, and 0.15 μg/ml) for 20 h before viability was assessed using the MTT assay. The MTT assay results were normalized to an untreated sample. *H*, viability in IECs left untreated (*0*), stimulated with poly(I:C) alone (2 μg/ml), transfected with poly(I:C) using Lipofectamine RNAimax (2 μg/ml), or treated with only Lipofectamine RNAimax for 43 h before the viability of the cells was assessed using the MTT assay. The MTT assay results were normalized to an untreated sample. The results show mean ± S.D. of five samples. All results are representative of at least two independent experiments.

CXCL10 is an interferon-inducible cytokine that is efficiently induced by IFNβ. Poly(I:C) is an effective inducer of IFNβ in many cell types, and type I interferons are known to inhibit proliferation, impair angiogenesis, and have apoptotic effects in several cancers (12). We therefore found it puzzling that the metastatic IECs displayed a potent CXCL10 response toward poly(I:C), in contrast to healthy cells and non-metastatic IECs. Nevertheless, we observed that poly(I:C) concentrations down to 0.3 μg/ml were sufficient to induce CXCL10 production in HT29 cells following 20 h of stimulation (Fig. 3*A*). Initial CXCL10 protein production was detected after 6 h of stimulation with poly(I:C) (2.5 μg/ml) and plateaued after 12 h of stimulation (Fig. 3*B*). It has been shown previously that IFNβ is induced in HT29 and SW480 cells in response to transfection with poly(I:C) by a mechanism dependent on the cytosolic RNA helicase RIG-I (37). In line with this report, we observed that all tested IECs induced IFNβ mRNA in response to transfection with poly(I:C) in complex with Lipofectamine RNAiMAX (LF) but failed to induce IFNβ in response to addition of poly(I:C) alone (Fig. 3*C*). IFNβ protein was also detected in the supernatants of HT29, SW620, and Caco-2 cells in response to transfected poly(I:C) but not in response to poly(I:C) addition (Fig. 3*D*). IFNα protein was not detected (<12.5 pg/ml) in the same supernatants when measured by ELISA (data not shown), signifying that IFNα is not produced in any of these IECs in response to poly(I:C) addition or transfection. HT29 cells were further stimulated with poly(I:C) at intervals between 3–24 h and assayed for IFNβ and CXCL10 mRNA to determine whether IFNβ is induced at earlier time points in these cells in response to poly(I:C) addition. Although CXCL10 induction was observed in these cells after 3–6 h of poly(I:C) addition, only a slight and transient induction of IFNβ mRNA was observed at 3 h of stimulation in HT29 cells (Fig. 3*E*). Poly(I:C) also failed to activate the IFNβ promoter in SW620 cells transfected with an IFNβ reporter luciferase, although marked IFNβ activation was observed in response to poly(I:C) transfection in these cells following 21 h of treatment (data not shown). These results show that metastatic IECs induce CXCL10 release but do not elicit IFNβ in response to poly(I:C) addition. This does not appear to be due to a defect in signaling leading to IFNβ because IFNβ induction was observed in these cells in response to poly(I:C) transfection. Thus, the mode of poly(I:C) delivery dictates the response induced in these IECs.

HT29 cells were pretreated with the protein synthesis inhibitor cycloheximide to verify that CXCL10 is induced directly in response to poly(I:C), independent of IFN signaling. The stimulation time (8 h) was chosen to be short enough to avoid to the toxic effects of cycloheximide but sufficient to observe CXCL10 mRNA expression and protein production in mock-treated cells stimulated with poly(I:C). We observed potent induction of CXCL10 mRNA, even in the presence of cycloheximide (Fig. 3 F), whereas CXCL10 protein expression was completely abrogated following cycloheximide treatment, as assessed by ELISA (data not shown). This implies that poly(I:C)-induced CXCL10 expression is a primary response in HT29 cells, occurring independently of IFNβ induction and signaling.

Poly(I:C) induces apoptosis in some cancer cells in a TLR3-dependent manner. High TLR3 expression in tumor cells has therefore been appreciated as a way to target these cells using poly(I:C). To test whether cancerous IECs were sensitive to poly(I:C) treatment, we initially assayed the viability of HT29 cells following exposure to poly(I:C) for 20 h using the MTT assay. The viability of these cells was unimpaired even after exposure to high concentrations of ligand (50 μg/ml) (Fig. 3*G*), indicating that these cells are highly resistant to the apoptotic effects of poly(I:C). The viability of IECs following poly(I:C) addition or poly(I:C) transfection was also compared. The viability of HT29, SW620, SW480, and Caco-2 cells was unaffected by addition of poly(I:C) but was markedly impaired in these IECs in response to transfected poly(I:C) (2 μg/ml) (Fig. 3*H*). HCT116 cells displayed some reduction in viability following exposure to poly(I:C) for 43 h but were considerably more sensitive to poly(I:C) transfection (Fig. 3*H*). Poly(I:C) transfection is therefore much more effective in inducing apoptosis in cancerous IECs than addition of poly(I:C) alone. This is in line with reports documenting the apoptotic effects of poly(I:C) upon transfection (12). Combined, these results demonstrate that the metastatic IECs SW620 and HT29 induce CXCL10 in response to poly(I:C) addition but fail to induce sustained IFNβ expression. These cells are also very resistant to the apoptotic effects of added poly(I:C).

### IRF3 is activated in HT29 cells in response to both poly(I:C) addition and transfection

**Figure 4.**
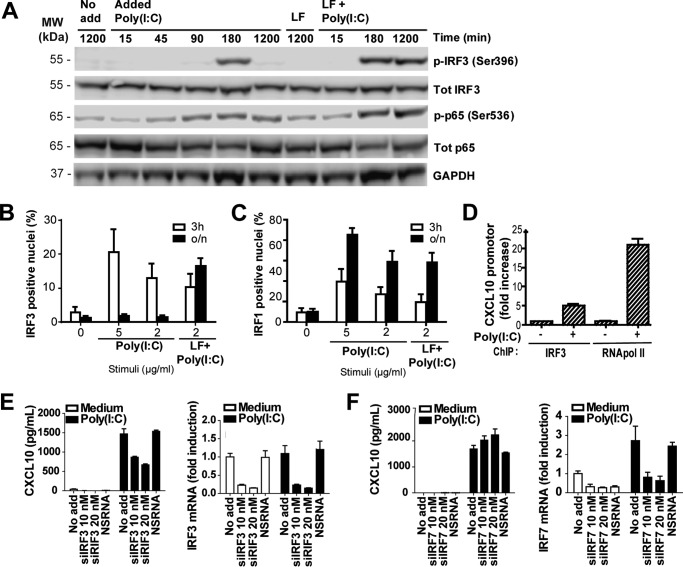
**IRF3 is phosphorylated, translocates to the nucleus, and binds the CXCL10 promoter in HT29 cells in response to addition of poly(I:C).** *A*, Western blots of HT29 cells were stimulated with poly(I:C) alone (*Poly(I:C)*, 2.5 μg/ml) or transfected with poly(I:C) using Lipofectamine RNAimax (*LF* + *Poly(I:C)*, 0–1200 min) and stained with antibodies against phospho-IRF3^Ser-396^, total IRF3, phosphor-p65^Ser-536^, total p65, or GAPDH. The results are representative of two independent experiments. *MW*, molecular weight. *B* and *C*, nuclear accumulation of IRF3 (*B*) and IRF1 (*C*) in HT29 cells left untreated (*0*), stimulated with poly(I:C) (5–2 μg/ml), or transfected with poly(I:C) complexed with Lipofectamine RNAimax (2 μg/ml) for 3 h or overnight (*o/n*). Stimulated cells were fixed and immunostained for IRF3 or IRF1, and cell nuclei were stained with Hoechst 3342. Cells were visualized by automated imaging, and analysis was done using ScanR. The results show the percentage of cells with positive staining of IRF3 and IRF1 in the nucleus. The results show mean ± S.D. of triplicate samples with a minimum of 1300 cells assayed and are representative of three independent experiments. *D*, CXCL10 promotor occupancy by IRF3 in HT29 cells after poly(I:C) (2 μg/ml) stimulation for 3 h. IRF3 binding to the CXCL10 promoter was investigated by ChIP followed by qPCR of the CXCL10 promoter region. RNA polymerase II occupancy was measured as a control. *E* and *F*, CXCL10 production (*left panels*) and IRF mRNA expression (*right panels*) in HT29 cells left untreated (*No add*), treated with siRNA against IRF3 (*E*) or IRF7 (*F*) (10 nm), NS RNA (10 nm), or transfection reagent alone (*LF*) for 24 h. Cells were subsequently stimulated with poly(I:C) (2.5 μg/ml) for 6 h. CXCL10 release was assessed by ELISA, whereas silencing of IRF3 and 7 was confirmed by assessing mRNA expression by qPCR using GAPDH as a reference control. The results show mean ± S.D. of triplicate samples.

IRF3 is a central transcription factor in driving the gene expression of IFNβ in response to poly(I:C) (38). We therefore assessed IRF3 activation in HT29 cells in response to poly(I:C) to determine differences in IRF3 activation following treatment with poly(I:C) alone or with poly(I:C) complexed with LF. Impaired IRF3 activation in response to poly(I:C) addition could potentially explain the absence of IFNβ induction in these cells. A hallmark of IRF3 activation is the phosphorylation of Ser-396 on IRF3 (39). Phosphorylation of this site was assayed in HT29 in response to poly(I:C) addition or poly(I:C) transfection by Western blotting. We observed IRF3 phosphorylation in HT29 cells both in response to poly(I:C) addition and poly(I:C) transfection after 180 min of stimulation, indicating that IRF3 is activated in response to both treatments (Fig. 4*A*). Persistent IRF3 phosphorylation was, however, observed in HT29 cells in response to transfected poly(I:C) after 1200 min but not in response to poly(I:C) addition (Fig. 4*A*). Similar results were obtained in SW620 cells (data not shown), which also induce CXCL10 but not IFNβ, in response to poly(I:C) addition (Figs. 1*B* and 3, *C* and *D*). Western blots of stimulated HT29 cells stained with anti-p65^Ser-536^ showed a similar degree of phosphorylation in response to addition or transfection of poly(I:C), indicating that p65 is efficiently phosphorylated at this site in response to both treatments (Fig. 4*A*). Thus, the absence of IFNβ response in HT29 in response to poly(I:C) addition does not appear to be due to failure to activate IRF3 or NF-κB, although more sustained phosphorylation of IRF3 was observed in response to transfected poly(I:C).

We further assessed nuclear localization of IRF3 in HT29 in response to poly(I:C) addition or poly(I:C) transfection. The transcription factor IRF1, which has also been implicated in IFNβ induction, was assayed in parallel. HT29 cells stimulated with poly(I:C) alone or transfected with poly(I:C) were stained with antibodies against IRF3 or IRF1 and visualized by high-throughput immunofluorescence microscopy. Nuclear localization of both IRF3 and IRF1 was observed in response to both poly(I:C) addition and poly(I:C) transfection following 3 h of treatment (Fig. 4, *B* and *C*), indicating that both transcription factors are activated in response to both addition and transfection of poly(I:C). Persistent nuclear localization of IRF3 was, however, observed after overnight stimulation in response to transfected poly(I:C) but not in response to added poly(I:C) (Fig. 4*B*). IRF1 expression in the nucleus persisted after overnight stimulation in response to both poly(I:C) addition and poly(I:C) transfection (Fig. 4*C*), indicating that the duration of IRF1 activation is similar in response to the two treatments. These results imply that IRF3 is activated in HT29 cells both in response to poly(I:C) addition and poly(I:C) transfection, although poly(I:C) transfection induces more persistent IRF3 activation, which, in turn, may be required for IFNβ production in these cells.

CXCL10 is an IFN-inducible protein but has also been shown to be a primary response (40) and can be triggered independent of type I IFN production in certain types of cells (41). Because our previous results indicated that poly(I:C)-induced CXCL10 production is a primary response in HT29 cells (Fig. 3*F*), we proceeded to determine whether IRF3 binds the CXCL10 promoter directly in HT29 cells in response to poly(I:C) addition. We did this by performing ChIP of HT29 cells stimulated with poly(I:C) for 3 h, using antibodies against IRF3 or RNA polymerase II. We then assessed CXCL10 promoter binding by qPCR. The ChIP analysis revealed that both IRF3 and RNA polymerase II are recruited to the CXCL10 promoter in HT29 cells in response to poly(I:C) addition (Fig. 4*D*), implying that IRF3 is directly involved in driving CXCL10 induction. The role for IRF3 in driving CXCL10 expression was verified by treating HT29 cells with siRNA against IRF3 prior to poly(I:C) stimulation. Silencing IRF3 in HT29 cells diminished CXCL10 production following poly(I:C) addition (Fig. 4*E*), demonstrating the importance of IRF3 in this response. IRF7 has also been implicated in driving CXCL10 production and type I IFNs in response to TLR activation (38). Normal induction of CXCL10 was, however, observed in response to poly(I:C) in IRF7-silenced HT29 cells (Fig. 4*F*). In summary, these results demonstrate that poly(I:C) addition induces activation and binding of IRF3 to the CXCL10 promoter in HT29 cells. Thus, the inability of these cells to express IFNβ expression in response to poly(I:C) addition is not due to impaired IRF3 activation.

### SW620 and HT29 cells elicit CXCL10 independent of poly(I:C) internalization

**Figure 5.**
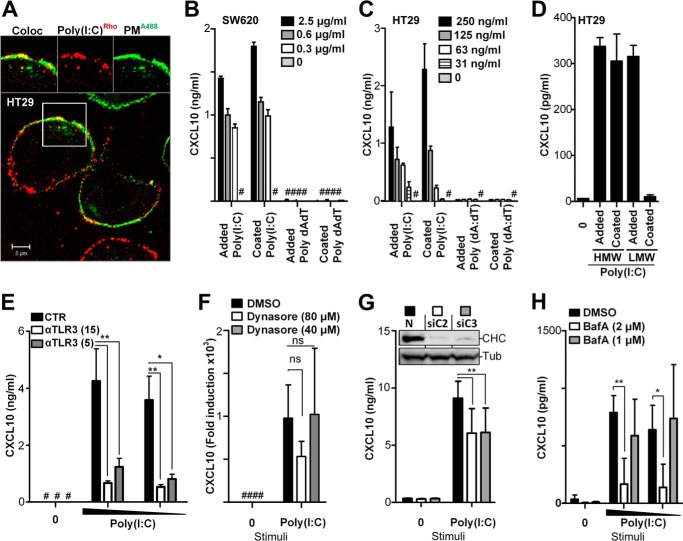
**CXCL10 production in metastatic IECs is elicited independent of poly(I:C) internalization but requires endosomal acidification.** *A*, confocal microscopy image of HT29 cells treated with poly(I:C)^Rhodamine^ (*red*, 5 μg/ml) for 2 h before cells were washed and fixed, and the plasma membrane (*PM*) was stained with an antibody against Na,K-ATPase (*PM^A488^*, *green*). Images (*top*) show co-localization (*Coloc*, *left*) and single tracks of poly(I:C)^Rhodamine^ (*center*) and plasma membrane PM^A488^ staining (*right*) of the area denoted by the *square* in the main image. *Scale bar* = 5 μm. *B* and *C*, SW620 (*B*) or HT29 (*C*) cells were treated with poly(I:C) (*Added Poly(I:C)*) or double-stranded DNA dA:dT (*Added dA:dT*) added in solution or by plating cells in wells precoated with poly(I:C) (*Coated Poly(I:C)*) or dA:dT (*Coated dA:dT*) with the given concentrations for 24 h before CXCL10 release was determined by ELISA. *D*, CXCL10 production in HT29 cells exposed to HMW poly(I:C) or LMW poly(I:C) (2 μg/ml) either added in solution (*Added*) or by plating cells in wells precoated with poly(I:C) (*Coated*) for 21 h. *E*, CXCL10 release in HT29 cells pretreated with anti-TLR3 (15 or 5 μg/ml) or control goat IgG (15 μg/ml) for 1 h prior to stimulation with poly(I:C) (5 or 2 μg/ml) for 10 h. CXCL10 content in the supernatant was assessed by ELISA. **, *p* < 0.01; *, *p* < 0.05 *versus* cells pretreated with control IgG (two-way ANOVA, Bonferroni post-test). The results in *A–D* show mean ± S.D. of triplicates and are representative of three independent experiments. #, below detection. *F*, CXCL10 expression in HT29 cells treated with the dynamin inhibitor Dynasore (80 or 40 μm) or the DMSO control for 30 min prior to stimulation with poly(I:C) (2.5 μg/ml) for 8 h. CXCL10 mRNA was determined by qPCR (normalized to medium control and the endogenous control TBP). The results show the mean of triplicates from three independent experiments ± S.D. *ns*, not significant (two-way ANOVA, Bonferroni post-test). #, below detection. *G*, CXCL10 production in HT29 cells transfected with NS RNA (20 nm) or two siRNAs against clathrin heavy chain 1 (siC2 and siC3, 10 + 10 nm) in two rounds for 26 h and 20 h prior to stimulation with poly(I:C) (2 μg/ml) for 8 h. The results show mean ± S.D. of triplicates and are representative of three independent experiments. **, *p* < 0.01 *versus* cells transfected with non-silencing siRNA (two-way ANOVA, Bonferroni post-test). *Inset*, Western blots of lysates of HT29 cells treated in parallel as described for siRNAs against clathrin heavy chain 1 (siC2 or siC3) or non-silencing RNA (*N*), stained with antibody against clathrin heavy chain 1 (*CHC*, *top blot*) or against α-tubulin (*Tubulin*, *bottom blot*). *H*, CXCL10 production in HT29 cells pretreated with bafilomycin A (2–1 μm) or the DMSO control for 30 min prior to stimulation with poly(I:C) (2 or 1 μg/ml) for 10 h. The supernatant was assayed for CXCL10 content by ELISA. The results show mean ± S.D. of triplicates and are representative of two independent experiments. **, *p* < 0.01; *, *p* < 0.05 *versus* untreated cells (two-way ANOVA, Bonferroni post-test).

Poly(I:C) is endocytosed by a clathrin-dependent pathway in mDC and HEK293 cells (20, 42). TLR3 is mainly expressed in the endoplasmic reticulum in resting cells but translocates to poly(I:C)-containing endosomes upon stimulation (20). TLR3 activation is demonstrated to occur in endolysosomal compartments in a process that requires acidification (25, 28, 43, 44). Using confocal microscopy, we observed that fluorescent poly(I:C) is endocytosed in IECs 1 h after addition (data not shown), but that poly(I:C) also remains bound to the plasma membrane, even after stimulation for 2 h (Fig. 5*A*). This is in line with previous observations in HEK293 cells (44). Because we observed TLR3-dependent CXCL10 induction in the metastatic cell lines SW620, HCT116, and HT29 upon addition of poly(I:C), we hypothesized that TLR3 might be present in the plasma membrane and may signal from the surface of these cells. Expression of TLR3 at the plasma membrane could potentially allow the receptor to interact with ligand independent of endocytosis. We initially tested this hypothesis by coating high-binding 96-well plates with poly(I:C) overnight. Separate wells were coated with double-stranded DNA poly(dA:dT) as a control. HT29 or SW620 cells were subsequently plated in wells coated with poly(I:C), poly(dA:dT), or PBS. Immobilization of poly(I:C) in the wells allows surface receptors of plated cells to interact with ligand but inhibits ligand internalization. HT29 and SW620 cells were also treated with poly(I:C) or poly(dA:dT) added in solution for comparison. Supernatant from HT29 and SW620 cells was harvested after 20 h of incubation and assayed for CXCL10 content by ELISA. Interestingly, immobilization of poly(I:C) still induced CXCL10 release in HT29 and SW620 cells (Fig. 5, *B* and *C*), indicating that internalization of ligand is not necessary for TLR3 activation and suggesting that TLR3 may be expressed on the surface of these cells. Using the same assay, we compared the high-molecular-weight (HMW) poly(I:C) (1.5–8 kb) with low-molecular-weight (LMW) poly(I:C) (0.2–1 kb) and found that coated HMW poly(I:C) induces robust CXCL10 release in HT29 cells, whereas coated LMW poly(I:C) failed to induce CXCL10 release in these cells (Fig. 5*D*). LMW poly(I:C) added in solution induced similar levels of CXCL10 release as HMW poly(I:C) (Fig. 5*D*). These results suggest that TLR3 is expressed in the plasma membrane and can be activated at the surface by long strands of poly(I:C), presumably by effectively multimerizing TLR3 as described previously (28). We further observed that pretreatment of HT29 cells with a polyclonal anti-TLR3 antibody impaired poly(I:C) induced CXCL10 release in these cells (Fig. 5*E*), further proposing that these cells express surface TLR3 and that signaling can be inhibited by blocking TLR3.

To verify that TLR3 signals from the cell surface, we pretreated HT29 cells with Dynasore, an inhibitor of receptor-mediated endocytosis, prior to poly(I:C) stimulation for 8 h. CXCL10 expression was subsequently determined by qPCR. We found that CXCL10 induction was not significantly impaired in poly(I:C)-stimulated HT29 cells upon inhibition of endocytosis with Dynasore (Fig. 5*F*), suggesting that receptor-mediated endocytosis is not crucial for stimulation of these cells. To further confirm that TLR3 signaling can occur independently of clathrin-mediated endocytosis, we transfected HT29 cells with two siRNAs (siC2 and siC3) against clathrin heavy chain 1 (CHC) prior to stimulation with poly(I:C) for 8 h. CXCL10 content in the cell supernatant was determined by ELISA. Knockdown efficiency of CHC following siRNA treatment was confirmed in HT29 lysate by staining Western blots with antibody against CHC. Although we achieved very efficient silencing of CHC (Fig. 5*G*, *inset*), we only observed a partial impairment in CXCL10 production in poly(I:C)-stimulated HT29 cells upon silencing of CHC (Fig. 5*G*), indicating that ligand internalization is not crucial for signaling in these cells. Combined, these results suggest that TLR3 signaling in response to poly(I:C) can occur independent of ligand endocytosis in these cells, although poly(I:C) internalization may be required for full CXCL10 induction.

We also assessed the role of endosomal and lysosomal maturation on TLR3 signaling in HT29 cells. Bafilomycin A1, an inhibitor of vacuolar-type H^+^-ATPase (V-ATPase), inhibits endosomal and lysosomal acidification and has been shown previously to inhibit TLR3 signaling in several types of cells (25, 28, 44, 45). HT29 cells pretreated with bafilomycin A prior to 10 h of poly(I:C) stimulation displayed impaired CXCL10 production (Fig. 5*H*). This indicates that acidification of these compartments is important for enhancing TLR3 signaling in these cells, although endocytosis does not appear to be an absolute requirement for signaling.

### SW620 and HT29 cells express both full-length and cleaved TLR3 in the plasma membrane, and UNC93B1 is involved in TLR3-mediated CXCL10 production

**Figure 6.**
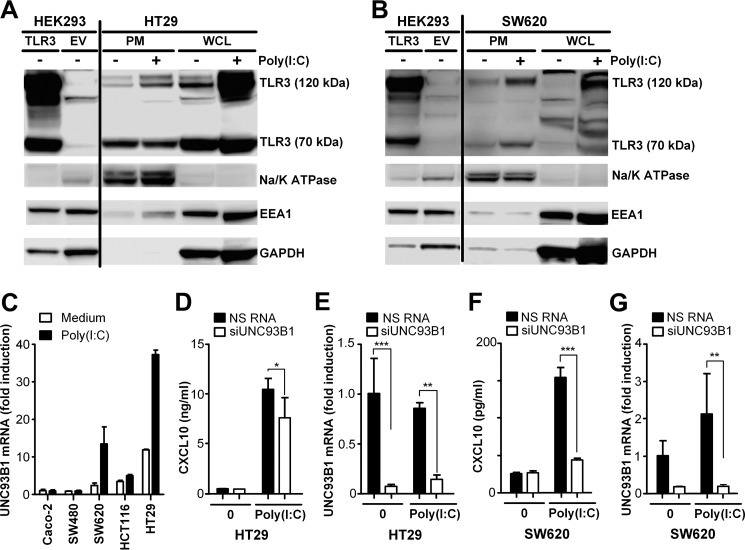
**TLR3 is expressed in the plasma membrane of metastatic IECs.** *A*, Western blot of whole-cell lysate of HEK293 cells transfected with TLR3 or empty vector (*EV*) or isolated plasma membrane fractions (*PM*) or whole cell lysate (*WCL*) from HT29 cells left untreated (−) or stimulated with poly(I:C) (+) (2.5 μg/ml) for 24 h. Blots were stained with antibodies against TLR3, Na,K-ATPase as a control for the plasma membrane protein fraction, the early endosome marker EEA-1, or GAPDH. *B*, Western blot of whole-cell lysate of HEK293 cells transfected with TLR3 or empty vector or isolated plasma membrane fractions or whole-cell lysate of SW620 cells left untreated (−) or stimulated with poly(I:C) (2.5 μg/ml) (+) for 24 h. Blots were stained with anti-TLR3, anti-Na,K-ATPase, anti-EEA1, or anti-GAPDH. *C*, UNC93B1 mRNA expression in Caco-2, SW480, SW620, HCT116, and HT29 cells treated with medium or poly(I:C) (5 μg/ml) for 24 h. UNC93B1 mRNA was determined by qPCR. The results were normalized to endogenous GAPDH expression and show -fold induction relative to the Caco-2 medium sample. The results are presented as mean ± S.D. of triplicates. *D* and *E*, CXCL10 production (*D*) and UNC93B1 (*E*) expression in HT29 cells treated with siRNA against UNC93B1 (20 nm) or NS RNA (20 nm) twice for 26 h and 20 h prior to stimulation with poly(I:C) (2 μg/ml) for 8 h. *F* and *G*, CXCL10 production (*F*) and UNC93B1 expression (*G*) in SW620 cells treated with siRNA against UNC93B1 (20 nm) or NS RNA (20 nm) in two rounds for 26 h and 20 h prior to stimulation with poly(I:C) (2 μg/ml) for 24 h. *D—G*, CXCL10 release in the cell supernatant was assessed by ELISA, whereas UNC93B1 mRNA expression in the cells was determined by qPCR using TBP as a reference control. The results show mean ± S.D. of triplicates and are representative of three independent experiments. ***, *p* < 0.001; **, *p* < 0.01; *, *p* < 0.05 *versus* NS RNA (two-way ANOVA, Bonferroni post-test).

The finding that poly(I:C) internalization does not seem to be essential for TLR3 signaling in SW620 and HT29 cells implied that TLR3 is expressed at the surface of these cells. Still, we also observed that CXCL10 production in response to poly(I:C) addition was strongly impaired following treatment with bafilomycin A, indicating that acidification of these compartments is required for TLR3 signaling in these IECs. TLR3 resides in the ER following synthesis and is transported via the Golgi to endosomes, where it is subject to proteolytic cleavage in a process dependent on acidification and cathepsins (24, 25, 27). Although SW620 and HT29 cells do not require ligand internalization to activate TLR3, the strong effect of bafilomycin A on poly(I:C)-induced CXCL10 production in these cells indicates that endosomal acidification is important for signaling. We reasoned that this could be due to impaired cleavage and/or transport of TLR3 to the cell surface as a result of endolysosomal neutralization. We proceeded to determine whether TLR3 was expressed in the plasma membrane of SW620 and HT29 cells and whether cleaved TLR3 is present at the surface of these cells. Expression of TLR3 in HT29 and SW620 cells is low and difficult to detect by immunofluorescence using commercial antibodies and appropriate blocking protocols. Plasma membrane fractions of lysates of unstimulated and poly(I:C)-stimulated SW620 and HT29 cells were therefore isolated, and we assayed TLR3 expression by Western blotting. Total and plasma membrane fractions of SW620 and HT29 cell lysates were separated by SDS-PAGE, and Western blots were stained using an antibody specific for TLR3. Na,K-ATPase staining was used as a positive control for the plasma membrane protein fraction, and early endosome marker Eea-1 staining was used as a control for the presence of endosomal membranes in the plasma membrane fraction. Whole-cell lysates of HEK293 cells transfected with TLR3 or an empty vector were also assayed as a positive control for TLR3 expression. Interestingly, we found both full-length (∼120 kD) and cleaved (∼70 kDa) TLR3 expressed in the plasma membrane fraction of both unstimulated and stimulated HT29 cells (Fig. 6*A*). Only full-length TLR3 appeared to be up-regulated in the plasma membrane fraction upon poly(I:C) stimulation, although we cannot exclude contamination of endosomal membranes in this fraction (Fig. 6*A*). Cleaved 70-kDa TLR3 was, however, clearly present in the plasma membrane of HT29 cells, both in the absence and presence of stimuli (Fig. 6*A*). Immunoblots of SW620 cells showed weak bands of both full-length and cleaved 70-kDa TLR3 in the plasma membrane fraction of SW620 cells (Fig. 6*B*), indicating that TLR3 is already present at the cell surface in unstimulated cells, although it is difficult to detect in whole-cell lysate (Fig. 6*B*). Strong up-regulation of both full-length and cleaved TLR3 was observed in the plasma membrane fraction of SW620 cells following poly(I:C) stimulation for 24 h (Fig. 6*B*). Eea-1 staining was minimal in the plasma membrane fractions from SW620 cells, demonstrating the purity of these plasma membrane fractions (Fig. 6*B*). These results show that these cells express both full-length and cleaved TLR3 in the plasma membrane, allowing TLR3 to sense extracellular dsRNA.

The chaperone protein UNC93B1 is required for transporting TLR3 from the ER to endosomes and is crucial for TLR3 signaling (21, 22). UNC93B1 is further required for proteolytic and posttranslational processing of TLR3 (24, 25) and has also been shown to be required for the transport of TLR3 to the cell surface in some cell types (25, 26, 46). The expression of both cleaved and full-length TLR3 in the plasma membrane of SW620 and HT29 cells suggested a role for UNC93B1 in TLR3 trafficking and signaling in these cells. We therefore assessed UNC93B1 expression in unstimulated and poly(I:C)-stimulated IECs by qPCR. The metastatic cell lines SW620 and HT29 markedly up-regulated UNC93B1 expression in response to poly(I:C) after 24 h of stimulation (Fig. 6*C*), whereas Caco-2 and SW480 cells failed to do so (Fig. 6*C*). This correlated with the ability of these IECs to induce CXLC10 in response to poly(I:C) (Fig. 1). We further determined the role of UNC93B1 in mediating poly(I:C)-induced CXCL10 production in HT29 cells by treating the cells with siRNA against UNC93B1 prior to stimulation with poly(I:C) for 8 h. SW620 cells were treated likewise but were stimulated with poly(I:C) for 24 h because these cells require a longer time to produce CXCL10 protein. CXCL10 in the supernatant was subsequently assessed by ELISA, whereas the remaining cells were assayed for UNC93B1 mRNA expression by qPCR to verify silencing. Treatment of HT29 cells with siRNA against UNC93B1 partially impaired CXCL10 production in response to poly(I:C) in these cells (Fig. 6*D*), suggesting that UNC93B1 is involved in TLR3 signaling in these cells (Fig. 6*E*) but not critical for CXCL10 production. In contrast, poly(I:C)-induced CXCL10 production in SW620 cells was greatly impaired following UNC93B1 silencing (Fig. 6, *F* and *G*), showing that TLR3 signaling in these cells is highly dependent on UNC93B1. Thus, UNC93B1 appears to play a role in TLR3 signaling in poly(I:C)-responsive IECs, but these cells display differences with regard to the extent to which they rely on UNC93B1. Although HT29 appear to be less dependent on UNC93B1, this transporter protein seems to be crucial for TLR3 signaling in SW620 cells. This, in turn, may be linked to differences in the abundance of cleaved TLR3 at the plasma membrane of these cells.

### Poly(I:C) stimulation increases the invasiveness of metastatic IECs in a TLR3-dependent manner

**Figure 7.**
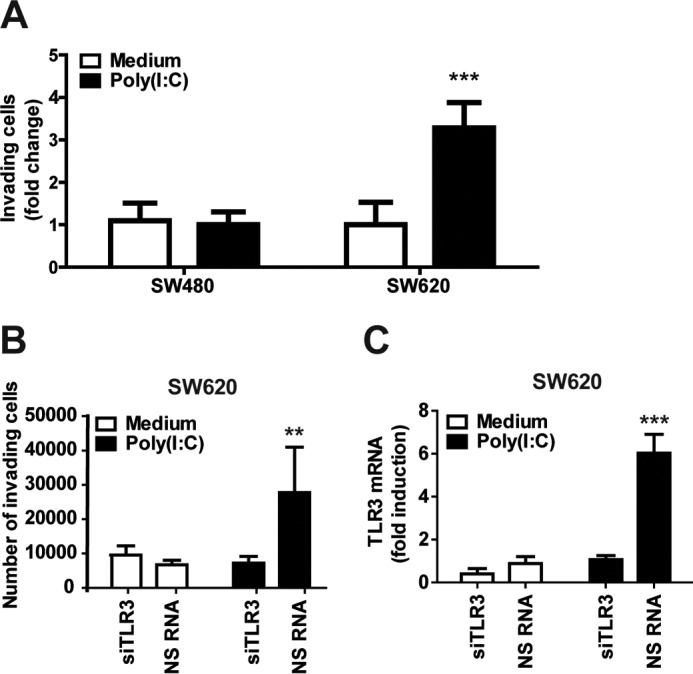
**Poly(I:C) stimulation enhances the invasive ability of SW620 cells in a TLR3-dependent manner.** *A* and *B*, SW620 and SW480 cells (*A*) or SW620 cells (*B*) transfected with silencing RNA against TLR3 (10 nm) or NS RNA were plated in a CytoSelect 96-well invasion plate and left untreated or stimulated with poly(I:C) (10 μg/ml) for 20 h. Cells that migrated through the membrane were lysed and quantified. Results were normalized to an untreated sample. *C*, SW620 treated with siRNA against TLR3 or NSRNA were assayed for TLR3 mRNA by qPCR to confirm gene silencing. -Fold change is shown relative to an untreated NS RNA sample. The results show mean ± S.D. of triplicates and are representative of three independent experiments. ***, *p* < 0.001; **, *p* < 0.01 *versus* medium or NS RNA treatment (two-way ANOVA, Bonferroni post-test).

Because we observed that metastatic IECs up-regulated TLR3 and responded to TLR3 stimulation, we proceeded to determine how TLR3 activation affects the invasive properties of metastatic IECs. The invasive capability of metastatic SW620 and non-metastatic SW480 cells following poly(I:C) treatment was determined using a transwell cell invasion assay. Cells were seeded into an upper chamber in serum-free medium and were either left untreated or stimulated with poly(I:C) for 20–24 h before assessment of the number of cells able to traverse an extracellular matrix–coated membrane. Interestingly, we found that the invasive ability of the metastatic cell line SW620 was up-regulated 3-fold following poly(I:C) stimulation, whereas SW480 cells were unaffected by poly(I:C) treatment in this assay (Fig. 7*A*). We further performed the same invasion assay using SW620 cells treated with siRNA targeting TLR3 to determine whether this effect was TLR3-dependent. Indeed, we found that the increased invasive ability of SW620 *cells* following poly(I:C) stimulation was highly dependent on TLR3, as the effect was abolished when this receptor was silenced (Fig. 7*B*). The efficiency of silencing TLR3 was verified by qPCR (Fig. 7*C*). These results indicate that triggering TLR3 can promote the invasive capability of metastatic IECs.

## Discussion

In this study, we report differences in the responsiveness of healthy and cancerous IECs to TLR ligands. In particular, we report key differences in TLR3 expression and responsiveness between non-metastatic and metastatic cancerous IECs and that TLR3 activation can enhance the invasive ability of these cells. Only highly metastatic IECs expressed detectable levels of TLR3 and induced the chemokine CXCL10 in response to poly(I:C) addition in a TLR3- and TRIF-dependent manner. Although the observed distinctions between IECs could be explained in terms of genetic variation, we observed interesting differences between SW480 and SW620 cells, which were originally isolated from the same donor, before and after tumor metastasis, respectively. Although SW480 cells did not express detectable levels of TLR3 and failed to respond to poly(I:C) addition, its metastatic counterpart, SW620 cells, up-regulated TLR3 and produced CXCL10 in a TLR3-dependent manner following poly(I:C) stimulation, suggesting that increased TLR3 expression could be a characteristic of progressed cancer. SW480 and SW620 cells share the same genetic background but display epigenetic differences (47) that appear to control the expression and regulation of TLR3. Although several malignant IECs are known to express functional TLR3, there are few reports of the expression and function of TLR3 in healthy primary IECs. TLR3 expression in the human intestinal epithelium from healthy individuals has been reported (48), but this has been mainly studied by immunohistochemistry. Our results suggest that increased TLR3 expression and TLR3-mediated CXCL10 induction is an attribute of cancer IECs rather than healthy IECs.

The majority of studies of the role of TLR3 in cancer have focused on applying dsRNA analogues in cancer therapy because of their ability to induce growth arrest and apoptosis in some TLR3-expressing tumors (9, 11, 49). The reported tumor-suppressive, anti-angiogenic, and apoptotic effects of TLR3 activation in the tumor microenvironment have predominantly been attributed to the induction of type I IFN and activation of effector cells that impair proliferation and induce apoptosis in tumor cells. However, in this study, activation of TLR3 in metastatic IECs by poly(I:C) did not induce IFNβ in metastatic IECs, and we did not observe decreased cell viability in response to poly(I:C) addition. Despite the lack of IFNβ production, we still observed induction of CXCL8 and CXCL10 in response to poly(I:C) addition in these cells, demonstrating that metastatic IECs are able to respond to poly(I:C) while avoiding the apoptotic effects associated with TLR3 activation. An unfortunate effect could then be that CXCL8 and CXCL10 display pro-tumor activity in the absence of tumor-inhibiting type I IFNs. Both CXCL8 and CXCL10 are chemoattractants that have been implicated in colorectal cancer progression (50, 51). Increased expression of CXCL8 and its receptor CXCR2 on tumors and in the tumor microenvironment are associated with increased tumor growth, angiogenesis, and metastatic potential of colon cancer cells (52–54). Although the chemokine CXCL10 is known to exert anti-angiogenic properties and mediate the recruitment of CXCR3-expressing mononuclear cells with anti-tumor activities, CXCL10 and its receptor CXCR3 are increasingly being recognized as pro-tumorigenic in several types of cancers, including CRC (55). Elevated serum CXCL10 and increased expression of CXCL10 and CXCR3 in tumor cells have been associated with a poor prognosis and metastasis (50, 51, 56–58), suggesting that the CXCL10–CXCR3 axis may promote CRC progression in some cases. We speculate that the induction of CXCL10 and CXCL8 upon TLR3 activation in metastatic IECs may drive the migration and invasiveness of these cells in the absence of IFNβ production. TLR signaling can also trigger changes in the expression of a number of adhesion molecules as well as up-regulate several matrix metalloproteases (MMPs) (59), which may promote the migration and invasiveness of IECs. Interestingly, TLR4 has been shown to enhance IEC adhesion and invasion by up-regulating the urokinase plasminogen activator (PLAU) and its receptor PLAUR, thereby activating the PLAU system (60). Increased expression of PLAUR and components of the plasminogen activation system correlate with malignancy and are associated with metastasis in several types of cancer, including colon cancer (61–64). Binding of PLAU to PLAUR leads to production of active plasmin, which facilitates activation of MMPs. TLRs induce several MMPs that may participate in the degradation of the extracellular matrix and promote invasion (59, 65). PLAUR and its ligand can also affect adhesion to the ECM and migration via interaction with integrins (64). Poly(I:C) also up-regulates PLAUR in a pharynx metastatic cell line, suggesting that TLR3 signaling in metastatic cells may promote cell migration and invasion by activating the PLAU system (66).

HT29 and SW480 cells have been shown to induce IFNβ in response to transfected poly(I:C) by a mechanism dependent on the cytosolic RNA helicase RIG-I but not in response to added poly(I:C) (37). This is in line with our observations. Introduction of poly(I:C) into the cytosol by transfection induces potent cell death in all the cancer IECs, demonstrating that the apoptotic effects of poly(I:C) on cancer cells is highly dependent on localization of poly(I:C) to the cytosol. IFNβ likely mediates the apoptotic effects of cytosolic poly(I:C). The reason why IECs fail to induce IFNβ in response to added poly(I:C) is unclear. The transcription factor IRF3 induces IFNβ downstream of both TLR3 and RIG-I. We clearly observed IRF3 activation in response to both poly(I:C) addition and transfection in HT29 cells, although previous reports show that IRF3 activation is only observed in response to transfected poly(I:C) in these cells (37). This discrepancy may be due to the sensitivity of the assays utilized by Hirata *et al.* (37); they observed IRF3 activation only at late time points after poly(I:C). In line with Hirata *et al.* (37), we also observed stronger and more prolonged IRF3 activation in response to transfected poly(I:C) in comparison with poly(I:C) addition alone.

Both the IFNβ and CXCL10 promoters are activated by cooperative binding of NF-κB p65 and IRF3 in several cell types (38, 41, 67, 68). The absence of an IFNβ response in metastatic IECs following poly(I:C) addition is not due to failure to activate IRF3 and does not appear to involve IRF7. Differential composition of NF-κB and IRF3 at the CXCL10 and IFNβ promoter could potentially permit CXCL10 induction without inducing IFNβ in response to added poly(I:C). NF-κB p65 is indeed required as a co-activator for IRF3 binding to the interferon-stimulated response element site in the CXCL10 promoter in response to LPS, whereas LPS-induced IFNβ production is driven by sequential binding of IRF3 and NF-κB p65 (38, 69, 70). Differential assembly of p65 and IRF3 at the CXCL10 and IFNβ promoter in metastatic IECs could explain the difference in induction of these genes in response to added poly(I:C). However, poly(I:C)-induced CXCL10 did not require the binding of p65 to IRF3 in previous studies (69, 70). The NF-κB regulatory domain in the IFNβ promoter that binds the p50–p50 homodimer may also play a role in restricting IFNβ induction in response to added poly(I:C). Notably, optimal IFNβ induction has also been shown to require binding of p65 beyond the enhanceosome in an essential cluster of homotypic κB sites 3′ downstream of the gene (71).

Because we see persistent IRF3 activation in response to transfected poly(I:C), we speculate that additional posttranslational modifications of IRF3 may be induced by the RIG-I pathway that are required for IFNβ induction. Additional co-activators may also be recruited to the IFNβ enhanceosome in response to poly(I:C) transfection. Abnormal expression of negative regulators that specifically target TLR3-mediated IFNβ induction in metastatic IECs could also explain the absence of IFNβ induction in IECs in response to added poly(I:C). A few negative regulators of TLR3-induced IFNβ have been described that permit TLR3-IRF3–mediated CXCL10 induction as well as RIG-I–induced IFNβ. One of these regulators is the bromodomain and extraterminal family member BRD4, which is essential for poly(I:C)-induced recruitment of IRF3 and c-Jun to the IFNβ promoter. Inhibitors of BRD4 impair IFNβ induction without affecting IRF3 phosphorylation or IRF3 nuclear localization (72). Repressed BRD4 expression in metastatic IECs may potentially explain the impaired IFNβ induction observed in these cells in response to added poly(I:C), particularly because it is not essential for production of IRF3-dependent chemokines. The adaptor molecule SARM is another candidate that impairs TRIF-dependent IFNβ and CCL5 induction (73). SARM is required for optimal polymerase II recruitment and assembly of transcription factors at the CCL5 promoter in response to LPS but does not affect CXCL10 induction or p65 or IRF3 translocation to the nucleus (74). Other candidates include the tripartite motif (TRIM) E3-ubiquitin ligase family members, which regulate many aspects of antiviral signaling (75). TRIM38 is particularly interesting because it has been suggested to be a negative regulator of TLR3–TRIF signaling but a positive regulator of RLR signaling (76). TRIM38 could therefore control the differential induction of IFNβ in response to added and transfected poly(I:C) in metastatic IECs. High TRIM38 expression could also explain the prolonged activation of IRF3 in response to poly(I:C) transfection in IECs.

Resting mDCs express TLR3 mainly in the ER, but TLR3 translocates and accumulates in poly(I:C)-containing endosomes upon stimulation (20). Endosomal TLR3 translocation is dependent on the transporter protein UNC93B1, and signaling occurs in a process that requires acidification (19, 22, 29, 30). Consequently, TLR3 signaling is considered to occur from endosomes. TLR3 expression and activation at the cell surface have been proposed in fibroblasts and bronchial epithelial cells (14, 17), indicating that signaling occurs from the plasma membrane in some cell types. Our results show that TLR3 is expressed in the plasma membrane of the metastatic IECs SW620 and HT29 and suggest that TLR3 signaling can occur from the cell surface in these cells. Pohar *et al.* (26, 44) demonstrated that overexpression of UNC93B1 leads to translocation of TLR3 to the cell surface in endothelial cells and epithelial cells overexpressing TLR3; however, the dynamin inhibitor Dynasore impaired TLR3-mediated IFNβ and NF-κB promoter activation in HEK293 cells upon TLR3 and UNC93B1 overexpression (44), and the authors conclude that endocytosis is still necessary for TLR3 signaling. Notably, their results show that Dynasore only partially affected IFNβ promoter activity in response to poly(I:C) stimulation (44), which suggests that signaling may in part occur from the plasma membrane. We observed that immobilization of poly(I:C) did not impair CXCL10 induction in SW620 or HT29 cells and that substantial TLR3-mediated CXCL10 induction occurred despite efficient inhibition of clathrin-dependent endocytosis, signifying that some signaling can occur independent of ligand-internalization in these cells.

Pohar *et al.* (44) further observed that bafilomycin A completely inhibited TLR3-mediated IFNβ and NF-κB promoter activation in HEK293 cells overexpressing TLR3, demonstrating the importance of endolysosomal acidification in TLR3 signaling. This is in line with a number of reports using bafilomycin A to document the importance of endolysosomal acidification in TLR3 signaling in several types of cells (25, 28, 45). We also observed that bafilomycin A significantly impaired CXCL10 production in HT29 cells in response to poly(I:C) addition. Thus, endosomal acidification is required for TLR3 signaling in these cells, although TLR3 signaling in metastatic IECs occurred largely independent of the endocytosis of poly(I:C) in these cells. Acidification is required for optimal binding of dsRNA to TLR3 (28, 45) and for cleavage of TLR3 in endosomes (24). The role of TLR3 cleavage is still unclear, and TLR3 cleavage may not be required for signaling (25). We observed both cleaved and full-length TLR3 at the plasma membrane of SW620 and HT29 cells prior to stimuli, implying that both processed and unprocessed TLR3 is present at the surface of resting cells. We propose that the profound effect of bafilomycin A on TLR3 signaling may be due to impaired TLR3 cleavage or impaired trafficking of TLR3 to the plasma membrane. Further studies are required to delineate whether TLR3 cleavage is required for signaling in these cells. Notably, bafilomycin is an inhibitor of V-ATPase, and some cancer cells express V-ATPases at the plasma membrane (77). Surface expression of V-ATPases in these cells is suggested to play a role in acidification of the tumor microenvironment, which, in turn, is associated with the metastatic potential of cancer cells (77). A possible mechanism is that the expression of V-ATPases in the plasma membrane of metastatic IECs may provide the acidic environment required for optimal binding of dsRNA to TLR3 (28, 45). This could perhaps explain the effects of bafilomycin A treatment on TLR3 signaling in these cells.

Our results show that expression of both cleaved and full-length TLR3 is present in the plasma membrane of the metastatic IECs SW620 and HT29. UNC93B1 has been shown to traffic TLR3 to the plasma membrane in some cell types (26, 44, 78), although it remains unknown whether TLR3 can signal from the cell surface in these cells. UNC93B1 knockdown nearly abolished TLR3-mediated CXCL10 induction in SW620 cells even after prolonged stimulation, implying a role for UNC93B1 in TLR3 signaling in these cells. In contrast, UNC93B1 only had a modest effect on TLR3-mediated signaling in HT29. This differential dependence on UNC93B1 may be linked to differences in UNC93B1 expression and the abundance of TLR3 at the plasma membrane in these cells in a resting state. We speculate that TLR3 signaling in SW620 cells may require UNC93B1 up-regulation to transport sufficient levels of TLR3 to the plasma membrane for optimal signaling. In contrast to SW620 cells, HT29 cells constitutively express UNC93B1 and high levels of TLR3 at the plasma membrane and may therefore be less dependent on UNC93B1 to traffic TLR3 to the plasma membrane.

The observation that TLR3 is expressed in the plasma membrane of metastatic epithelial cells and that TLR3-dependent CXCL10 induction can occur in response to extracellular stimuli suggests that malignant TLR3-expressing IECs could potentially respond to TLR3 ligands in the tumor microenvironment. Small nuclear RNA from tumor-derived exosomes has recently been demonstrated to activate TLR3 on lung epithelial cells (79). A number of reports also show that TLR3 can recognize endogenous host RNA released during cell death and damage (3, 4, 80, 81). Extensive tumor necrosis is observed in solid tumors such as CRC, and tumor necrosis correlates with disease progression in these tumors (82). Host RNA associated with necrotic cells and tumor-derived exosomes could potentially activate and up-regulate TLR3. The intratumor microenvironment of solid tumors is also intrinsically acidic (pH < 7) because of accumulation of lactic acid (83). This could provide an ideal environment to promote the binding of dsRNA to TLR3 and activation of this receptor, which is optimal between pH 5.5 and 6.5 (28, 45). Further studies are required to determine whether tumor necrosis in colorectal cancer tumors can activate TLR3 as well as determine the consequence of such activation on tumor progression. Our results indicate that some malignant IECs may up-regulate and express TLR3 at the cell surface, allowing it to be activated by extracellular stimuli, and that this increases the invasive ability of these cells. TLR3 activation culminates in the production of chemokines like CXCL10, but not IFNβ, in these cells. The consequence of this response in cancer progression has yet to be determined but appears to be an attribute of metastatic IECs and may promote the invasive ability of these cells. In conclusion, our results provide new insight into the expression and function of TLR3 in malignant IECs and suggest that altered TLR3 expression and signaling may have consequences for disease progression.

## Experimental procedures

### Cell lines and cell culture conditions

All cell lines were of human origin. The intestinal epithelial cancer cell lines Caco-2 (catalog no. HTB-37), SW480 (catalog no. CCL-228), HCT116 (catalog no. CCL-247), SW620 (catalog no. CCL-227), and HT29 (catalog no. HTB-38) and HEK293 cells were obtained from the ATCC. All IEC lines were cultivated in RPMI 1640 supplemented with 10% FCS, 2 mm glutamine, and 0.05% gensumycin in a humidified atmosphere of 5% CO_2_ at 37 °C. HEK293 cells were cultured in DMEM supplemented with 10% FCS, 2 mm glutamine, and 0.05% gensumycin in 8% CO_2_ at 37 °C. The FHC cell line was established from fetal colon tissue and purchased from the ATCC (catalog no. CRL-1831). FHC cells were cultured in DMEM:F12 supplemented with 10% FCS, cholera toxin (10 ng/ml), transferrin (5 μg/ml), hydrocortisone (100 ng/ml), and 10 mm HEPES in a humidified atmosphere of 37 °C and 5% CO_2_.

### Ligands

The synthetic lipopeptides Pam_3_Cys-Ser-Lys_4_ (P_3_C) and fibroblast-stimulating lipopeptide 1 (FSL-1) were from EMC (Tübingen, Germany). LPS from *Escherichia coli* O111:B4, MDP, and poly(I:C) were purchased from Invivogen (San Diego, CA). Vaccigrade poly(I:C) (Invivogen, vac-pic) was used in most experiments. HMW (Invivogen, tlrl-pic) and LMW poly(I:C) (Invivogen, tlrl-picw) were used for comparison of high- and low-molecular-weight poly(I:C). Recombinant TNF was purchased from Peprotech (Rocky Hill, NJ).

### Stimulation of cells

HT29, SW620, HCT116, SW480, Caco-2, and FHC cells were treated with medium, P_3_C (100 ng/ml), poly(I:C) (50 μg/ml), LPS (100 ng/ml), FSL-1 (100 ng/ml), MDP (1 μg/ml), or TNF (100 ng/ml) for 20 h.

#### 

##### Poly(I:C) stimulation

IECs were stimulated with poly(I:C) by adding ligand (0.15–50 μg/ml) to cells or transfecting ligand into cells by complexing poly(I:C) (1–2 μg/ml) with Lipofectamine RNAiMAX (1 μg:1 μl ratio) in RPMI for 15 min prior to addition to cells. Cells were treated for 8–24 h.

##### TLR3 blocking experiments

HT29 cells were pretreated with polyclonal anti-TLR3 antibody (R&D Systems, AF1487, 15 or 5 μg/ml) or control goat IgG (R&D Systems, AB-108-C, 15 μg/ml) for 1 h before stimulation with poly(I:C) (2 or 5 μg/ml) for 10 h.

##### Inhibitor experiments

Cells were pretreated with the dynamin inhibitor Dynasore (Sigma, 40 or 80 μm), cycloheximide (Sigma, 15 or 30 μg/ml), bafilomycin A (Sigma, 2 or 1 μm), or DMSO control for 30 min prior to addition of poly(I:C) stimuli.

### Cytokine measurements

Cells were stimulated for 8–24 h as indicated in the figure legends before the supernatant was assessed for CXCL8 (R&D Systems, DY008), CXCL10 (R&D Systems, DY266), or IFNβ (R&D Systems, 41410-1) by ELISA (R&D Systems) according to the protocol of the manufacturer.

### RNA interference

#### 

##### Silencing of TLR3 and TRIF

SW620 or HT29 cells were transfected for 24–48 h with 10–20 nm siRNA against TLR3 (TLR3_5, Qiagen, SI02630768; TLR3_8, Qiagen, SI02655156) or non-silencing control siRNA (Qiagen, SI03650325) using Lipofectamine RNAiMAX (Invitrogen) for TLR3 knockdown or with siRNA against TICAM-1/TRIF (Ambion, s45115) for TICAM/TRIF knockdown using Lipofectamine RNAiMAX. siRNA and transfection reagent (ratio, 1:2) were preincubated for 15 min in RPMI medium before being added to newly seeded cells. Knockdown of targets were confirmed in cell lysates by qPCR.

##### Silencing of IRF3 and IRF7

HT29 cells were transfected for 24 h with 10 nm siRNA against IRF3 (IRF3.4, Qiagen, SI02626526) or IRF7.1 (SI00448672) or non-silencing control siRNA (Qiagen, SI03650325) using Lipofectamine RNAiMAX (Invitrogen). siRNA and transfection reagent (ratio 1:2) were preincubated for 15 min in RPMI before being added to newly seeded cells. Knockdown of targets was confirmed by qPCR.

##### Silencing of CHC 1 and UNC93B1

CHC and UNC93B1 were silenced in HT29 or SW620 cells by complexing siRNAs (20 nm) with siLentFect (Bio-Rad) (ratio, 1:3) in Opti-MEM (Invitrogen) for 30 min before cells in solution were added to the transfection mixture and plated in 24-well plates. After 26 h of transfection, cells were treated with a new transfection mixture and incubated for an additional 20 h before stimulation experiments. To silence CHC, HT29 cells were transfected with non-silencing RNA (ON-TARGETplus NON-targeting Pool, Dharmacon, D-001810-10-05, 20 nm) or siRNA against CHC 2 (GCAATGAGCTGTTTGAAGA) or CHC 3 (TGACAAAGGTGGATAAATT) (Dharmacon, LQ-004001-00, 20 nm). To determine CHC protein knockdown, HT29 cells were lysed and assayed by Western blotting using an antibody against clathrin heavy chain 1 (X22, ABR Affinity Bioreagents, MA1-065) or α-tubulin (Santa Cruz Biotechnology, sc5286) as an endogenous control. To silence UNC93B1, cells were transfected with negative control siRNA (Qiagen, SI03650325, 20 nm) or a pool of UNC93B1_4 and UNC93B_6 FlexiTube siRNA (SI00756252 and SI04307912, Qiagen) (10 + 10 nm). Knockdown of UNC93B1 was confirmed in cell lysates by qPCR.

### RNA isolation, cDNA synthesis, and real-time qPCR

Total RNA was extracted using the NucleoSpin 96 RNA kit (Macherey-Nagel), and reverse transcription of RNA to cDNA was performed using the Applied Biosystems high-capacity RNA-to-cDNA kit following protocols of the manufacturer. The purity and concentration of RNA were determined using NanoDrop (Thermo Fischer Scientific). Real-time thermal cycling was performed with Applied Biosystems StepOnePlus^TM^. Perfecta qPCR FastMix^TM^ from Quanta and TaqMan probes (Life Technologies) for TLR3 (Hs01551078_m1), IFNβ (Hs01077958_s1), CXCL10 (Hs01124251_g1), UNC93B1 (Hs00276771_m1), IRF3 (Hs01547283_m1), IRF7 (Hs01014809_g1), TBP (TATA-box-binding protein) (Hs 00427620_m1), and GAPDH (Hs99999905_m1) were used. Gene expression data were analyzed using a generalized version of the comparative cycle threshold (CT) method for relative quantification with normalization to expression of the reference genes GAPDH or TBP.

### SDS-PAGE and Western blotting

HT29 or SW620 cells were cultured with or without poly(I:C) alone or poly(I:C) complexed with Lipofectamine RNAiMAX (Invitrogen). HEK293 cells were transfected for 24–48 h with pcDNA3 (Invitrogen) or TLR3^FLAG^ (Addgene, 13084) as a control for TLR3 expression using GeneJuice (Novagen) according to the instructions of the manufacturer. Cells were lysed, and gel electrophoresis was performed with the NuPAGE system using 10% BisTris gels (Invitrogen) following the protocol of the manufacturer. Proteins were transferred from gels to nitrocellulose membranes using the iBlot blotting system (Invitrogen). Membranes were blocked in 5% BSA in 0.01 %Tween 20/Tris-buffered saline (TBS-T) for 1 h at room temperature and stained with primary antibodies at 4 °C for 24–72 h in 5% BSA in TBS-T. Anti-TLR3 (Cell Signaling Technology, 6961) was used to determine TLR3 expression, and anti-GAPDH (Abcam, ab8245) was used as a loading control. Antibodies against IRF3 (Cell Signaling Technology, 11904) and phospho-IRF3^Ser-396^ (Cell Signaling Technology, 4947) were used to determine IRF3 activation in IECs upon poly(I:C) treatment, and antibodies against NF-κB p65 (Cell Signaling Technology, 8242) and phospho-NF-κB p65^Ser-536^ (Cell Signaling Technology, 3033) were used to determine activation of NF-κB in response to poly(I:C) stimulation. Isolation of plasma membrane proteins was performed using the Minute Plasma Membrane Protein Isolation Kit (Invent Biotechnologies) according to the manufacturer's protocol The resultant protein pellets were dissolved in lysis buffer. Anti-Na,K-ATPase (Abcam, ab7671) was used as a positive control for plasma membrane protein fractions, whereas an antibody against early endosome antigen 1 (EEA1) (Santa Cruz Biotechnology, sc-33585) was used as a marker for the presence of endosomal membranes. Anti-clathrin heavy chain 1 (X22, ABR Affinity Bioreagents, MA1-065) was used to determine expression and confirm knockdown of clathrin heavy chain 1 after siRNA treatment, and α-tubulin (Santa Cruz Biotechnology, sc5286) was used as a loading control in these experiments. Blots were stained with secondary HRP-conjugated anti-rabbit or anti-mouse antibody (P0448 and PO447, respectively; Dako) for 1 h, developed with Super Signal West Femto substrate (Pierce), and imaged using the Odyssey Fc system (Li-Cor).

### Viability assays

Cell viability was determined by MTT assay or using the Cell Titer Glo assay (Promega) according to the instructions of the manufacturer. For the MTT assay, cells were cultured in medium with 3-(4,5-dimethylthiazol-2-yl)-2,5-diphenyltetrazolium bromide (MTT, 0.5 mg/ml, Sigma) for 3 h before converted dye was solubilized in alkaline isopropanol with 0.25% 1 m NH_3_OH on a shaker for 30 min. The optical density at 570 nm (MTT assay) or luminescence (Cell Titer Glo assay) was measured using the Walla Victor^TM^3 1420 multilabel counter (PerkinElmer Life Sciences).

### Microscopy

HT29 cells were added to Rhodamine-labeled poly(I:C) (Invivogen) for 2 h before cells were washed and fixed with ice-cold fixation buffer (R&D Systems) for 10 min at room temperature. Cells were subsequently stained with antibody against Na,K-ATPase (Abcam, ab7671) for 1 h at 4 °C before staining with A488-conjugated anti-mouse secondary antibody (Invitrogen) for 30 min at 4 °C. Cells were observed with an Axiovert 100-M inverted confocal microscope equipped with an LSM 510 laser-scanning unit and a ×63 1.4 Plan Apochromat oil immersion objective (Zeiss, Jena, Germany).

In nuclear translocation studies, HT29 cells were seeded in 96-well glass-bottom plates (P96-1.5H-N, In Vitro Scientific, Sunnyvale, CA) and treated with poly(I:C) alone or complexed with Lipofectamine RNAiMAX for 3 h or overnight (15–24 h) before intracellular staining for IRF1 or IRF3 (84). In brief, cells were fixed with 2% paraformaldehyde in PBS on ice, permeabilized with PEM buffer (100 mm K-Pipes (pH 6.8), 5 mm EGTA, 2 mm MgCl_2_, and 0.05% saponin), quenched of free aldehyde groups with 50 mm NH_4_Cl in PBS with 0.05% saponin (PBS-S), and blocked with 20% human serum (HS) in PBS-S. Cells were stained with antibodies against IRF3 (D83B9, Cell Signaling Technology, 4302) or IRF1 (D5E4, Cell Signaling Technology, 8478) (1:200 dilution) in 1% HS/PBS-S overnight at 4 °C, stained with A647-labeled secondary antibody (Invitrogen, 2 μg/ml) for 15 min, and post-fixed with 4% paraformaldehyde/PBS. Nuclei were stained with Hoechst 3342 (200 ng/ml) in PBS-S. Automated imaging was done with the ScanR system (Olympus) using a ×20 objective, 1-s exposure time. 30 frames were captured for each well (∼1000–2000 cells) performed in triplicate. Automated image analysis was done with the ScanR software v1.3.0.3.

### ChIP

CXCL10 promoter occupancy by IRF3 was investigated in HT29 cells stimulated with poly(I:C) (2 μg/ml) for 3 h by ChIP as described previously (84). The antibodies used were anti-IRF3 (Santa Cruz Biotechnology, sc-9082X) or anti-RNA polymerase II (Millipore, clone CTD4H8), (4 μg/reaction). Antibodies were precipitated by protein G Dynabeads (Thermo Fisher, 110003D, 20 μl/reaction). Comparative promoter occupancy was determined by qPCR using the 2^−ΔΔCT^ method (85) with PowerUp SYBR Green (Thermo Fisher, #25780) using the primer sequences given in Ref. 86 (IP10 forward, TTTGGAAAGTGAAACCTAATTCA; IP10 reverse, AAAACCTGCTGGCTGTTCCTG).

### Ligand immobilization

Coating experiments were performed to determine whether SW620 and HT29 cells elicit CXCL10 independent of poly(I:C) internalization. 96-well plates (Corning, 3361) were coated overnight at 4 °C with PBS or titrations of poly(I:C) or double-stranded DNA poly(dA:dT) and then washed. SW620 or HT29 cells were plated (20,000 cells/well) in coated or uncoated wells. Cells in uncoated wells were stimulated by adding titrations of poly(I:C) or poly(dA:dT) in solution. Coating experiments comparing HMW poly(I:C) with LMW poly(I:C) were conducted in the same way.

### Transwell cell invasion assay

Cell invasion was determined with the CytoSelect^TM^ 96-well cell invasion assay (Cell Biolabs), using polycarbonate membrane transwell inserts (8-μm pore size); the upper surface of the insert membranes was coated with a layer of dried basement membrane matrix solution to discriminate between invasive and non-invasive cells. The assay was performed according to the instructions of the manufacturer. Briefly, siRNA or untreated cells were serum-starved overnight before cells were seeded into the upper chamber in serum-free medium (20,000 cells/well). Bottom wells were filled with 150 μl of RPMI medium supplemented with 10% FCS. The cells were either left untreated or treated with poly(I:C) (10 μg/ml) and incubated for 20–24 h at 37 °C and 5% CO_2_. The inserts were then placed in detachment buffer for 30 min to dissociate the invasive cells from the membrane. Upon detachment, cells were lysed and quantified using CyQuant GR fluorescent dye. The fluorescence of each well was determined at 480 nm/520 nm using the fluorescent plate reader function of the Walla Victor3^TM^ 1420 multilabel counter (PerkinElmer Life Sciences).

### Statistics

Statistical analysis was performed with GraphPad Prism version 5 or 6. The differences between two groups were determined by two-tailed Student's *t* test. Two or more groups were compared with one-way ANOVA with Holm-Sidak post-test or two-way ANOVA with Bonferroni post-test for multiple testing, as indicated in the figure legends.

## Author contributions

M. B. designed and conducted the majority of the experiments, analyzed and interpreted the results, and participated in writing the manuscript. B. B. designed and conducted the ChIP experiment shown in Fig. 4 and interpreted the results. O. K. E. and H. S. conducted and analyzed the experiments shown in Figs. 4–6 in collaboration with M. B. I. F. K. provided technical assistance and contributed to the experiments shown in Figs. 4 and 6. J. S. contributed to performing and analyzing the ScanR experiments shown in Fig. 4. T. E. contributed to the conception of the project. N. J. N. conceived the study, conducted and analyzed the experiments, and wrote the manuscript.

## Footnotes

3 The abbreviations used are:

TLR: Toll-like receptor
NLR: Nod-like receptor
RIG: retinoic acid–inducible gene
RLR: retinoic acid–inducible gene 1–like receptor
PRR: pattern recognition receptor
dsRNA: double-stranded RNA
mDC: myeloid dendritic cell
TIR: Toll/IL-1-resistance
TRIF: TIR domain–containing adaptor-inducing interferon β
ER: endoplasmic reticulum
IEC: intestinal epithelial cell
P_3_C: Pam_3_Cys-Ser-Lys_4_
MDP: muramyl dipeptide
qPCR: quantitative real-time PCR
NS: non-silencing
LF: Lipofectamine RNAiMAX
MTT: 3-(4,5-dimethylthiazol-2-yl)-2,5-diphenyltetrazolium bromide
HMW: high-molecular-weight
LMW: low-molecular-weight
CHC: clathrin heavy chain
V-ATPase: vacuolar-type H^+^-ATPase
MMP: matrix metalloproteinase
PLAU: urokinase plasminogen activator
PLAUR: urokinase plasminogen activator receptor
TRIM: tripartite motif
HS: human serum
ANOVA: analysis of variance.

## Note added in proof

In the version of this article that was published as a Paper in Press on July 17, 2017, an incorrect Na/K ATPase immunoblot was used in Fig. 6*A*. This error has now been corrected and does not affect the results or conclusions of this work.
